# Correction: Fecal microbiome of periparturient dairy cattle and associations with the onset of *Salmonella* shedding

**DOI:** 10.1371/journal.pone.0212528

**Published:** 2019-02-13

**Authors:** The PLOS ONE Staff

## Notice of republication

This article was republished on February 06, 2019 to correct for errors in the Data Availability statement introduced during the typesetting process. The publisher apologizes for the errors. Please download this article again to view the correct version. The originally published, uncorrected article and the republished, corrected article are provided here for reference.

## Supporting information

S1 File Originally published, uncorrected article. (PDF)

S2 File Republished, corrected article. (PDF)
