# Supplementary figures and images for: Fecal microbiome of periparturient dairy cattle and associations with the onset of Salmonella shedding
Source: PLoS One. 2018 May 11;13(5):e0196171. doi: 10.1371/journal.pone.0196171 (PMC5947886; doi:10.1371/journal.pone.0196171)

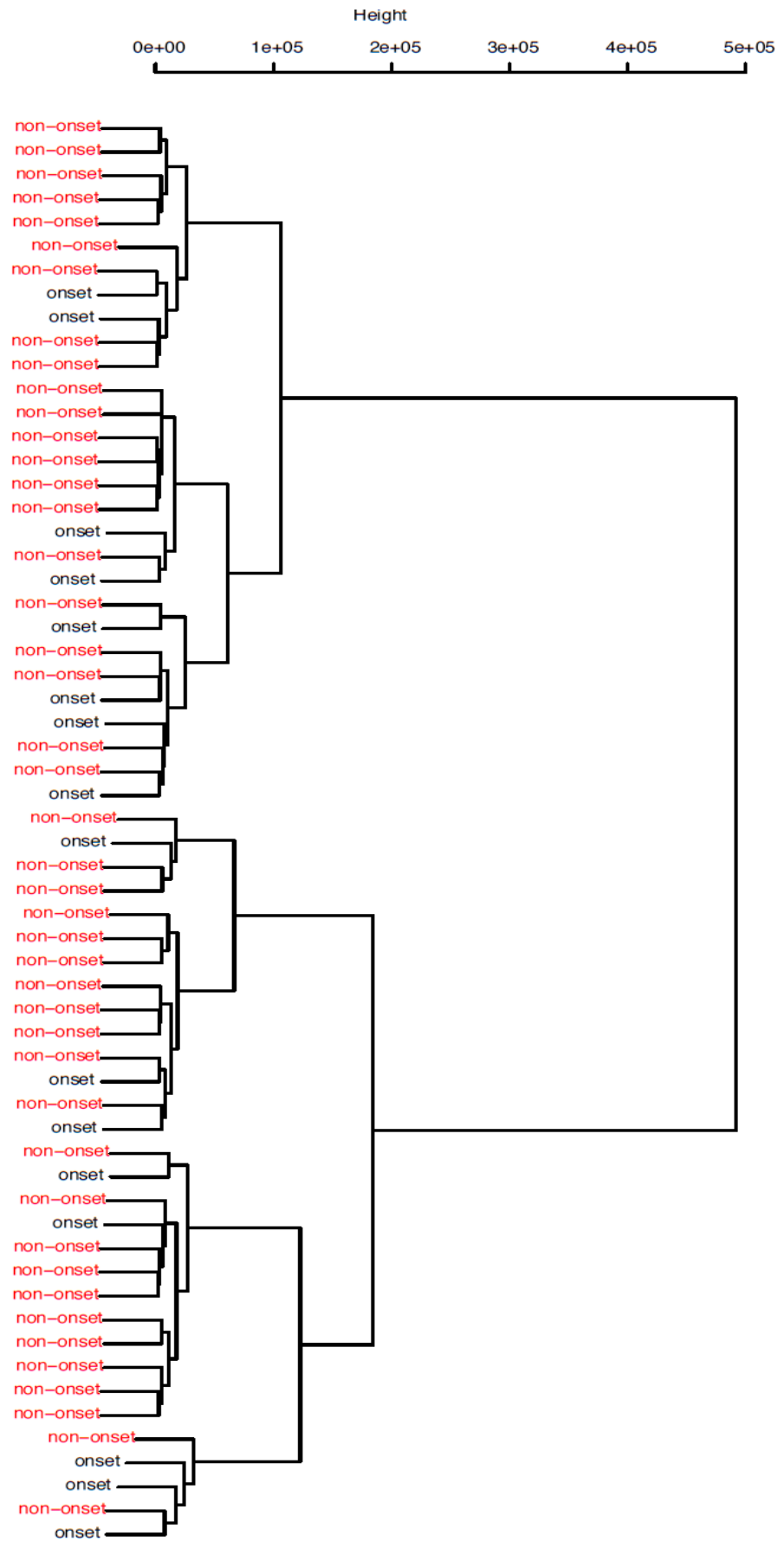

Supplement: S1 Fig — Non distinct clusters between bacterial communities of cows that had onset and non-onset were observed. (PDF) [file pone.0196171.s001.pdf]
