# Supplementary material for: Correction: Fecal microbiome of periparturient dairy cattle and associations with the onset of Salmonella shedding
Source: PLoS One. 2019 Feb 13;14(2):e0212528. doi: 10.1371/journal.pone.0212528 (PMC6373964; doi:10.1371/journal.pone.0212528)
Supplement: S2 File — (PDF) [file pone.0212528.s002.pdf]

RESEARCH ARTICLE

Fecal microbiome of periparturient dairy cattle and associations with the onset of *Salmonella* shedding

Lohendy Muñoz-Vargas[1]*, Stephen O. Opiyo[2], Rose Digianantonio[1¤a], Michele L. Williams[2¤b], Asela Wijeratne[2], Gregory Habing[1]*

1 Department of Veterinary Preventive Medicine, College of Veterinary Medicine, The Ohio State University, Columbus, Ohio, United States of America, **2** Ohio Agricultural Research and Development Center-Molecular and Cellular Imaging Center, The Ohio State University, Wooster, Ohio, United States of America

¤a Current address: College of Veterinary Medicine, Colorado State University, Fort Collins, Colorado, United States of America
¤b Current address: Department of Biology and Marine Science, Jacksonville University, Jacksonville, Florida, United States of America
* habing.4@osu.edu (GH); lohendymv@gmail.com (LMV)

OPEN ACCESS

Citation: Muñoz-Vargas L, Opiyo SO, Digianantonio R, Williams ML, Wijeratne A, Habing G (2018) Fecal microbiome of periparturient dairy cattle and associations with the onset of *Salmonella* shedding. PLoS ONE 13(5): e0196171. https://doi.org/10.1371/journal.pone.0196171

Editor: Arda Yildirim, Gaziosmanpasa University, TURKEY

Received: October 17, 2017

Accepted: April 6, 2018

Published: May 11, 2018

Copyright: © 2018 MuÃoz-Vargas et al. This is an open access article distributed under the terms of the Creative Commons Attribution License, which permits unrestricted use, distribution, and reproduction in any medium, provided the original author and source are credited.

Data Availability Statement: All relevant data are within the paper and its Supporting Information files. Sequencing data has been uploaded to a stable public repository: the European Nucleotide Archive (ENA), study accession number PRJEB25138, and the study unique name is ena-STUDY-THE OHIO STATE UNIVERSITY-22-02-2018-14:34:29:576-109.

Funding: This study was supported by the Intramural Unites States Department of Agriculture Formula Grant in the College of Veterinary Medicine

Abstract

Non-typhoidal *Salmonella enterica* is a zoonotic pathogen with critical importance in animal and public health. The persistence of *Salmonella* on farms affects animal productivity and health, and represents a risk for food safety. The intestinal microbiota plays a fundamental role in the colonization and invasion of this ubiquitous microorganism. To overcome the colonization resistance imparted by the gut microbiome, *Salmonella* uses invasion strategies and the host inflammatory response to survive, proliferate, and establish infections with diverse clinical manifestations. Cattle serve as reservoirs of *Salmonella*, and periparturient cows have high prevalence of *Salmonella* shedding; however, little is known about the association between the gut microbiome and the onset of *Salmonella* shedding during the periparturient period. Thus, the objective of this study was to assess the association between changes in bacterial communities and the onset of *Salmonella* shedding in cattle approaching parturition. In a prospective cohort study, fecal samples from 98 dairy cows originating from four different farms were collected at four time points relative to calving (-3 wks, -1 wk, +1 wk, +3 wks). All 392 samples were cultured for *Salmonella*. Sequencing of the V4 region of the *16S rRNA* gene using the Illumina platform was completed to evaluate the fecal microbiome in a selected sample subset. Analyses of microbial composition, diversity, and structure were performed according to time points, farm, and *Salmonella* onset status. Individual cow fecal microbiomes, predominated by Bacteroidetes, Firmicutes, Spirochaetes, and Proteobacteria phyla, significantly changed before and after parturition. Microbial communities from different farms were distinguishable based on multivariate analysis. Although there were significant differences in some bacterial taxa between *Salmonella* positive and negative samples, our results did not identify differences in the fecal microbial diversity or structure for cows with and without the onset of *Salmonella* shedding. These data suggest that determinants other than the significant changes in

at The Ohio State University. The grant was awarded to Dr. Greg Habing at the Department of Veterinary Preventive Medicine. The funder had no role in study design, data collection and analysis, decision to publish, or preparation of the manuscript.

Competing interests: The authors have declared that no competing interests exist.

the fecal microbiome influence the periparturient onset of *Salmonella* shedding in dairy cattle.

Introduction

Non-typhoidal *Salmonella enterica* (subsequently referred to as *Salmonella)* is a leading bacterial cause of foodborne illnesses worldwide [1]. Symptoms in affected individuals range from mild gastroenteritis to severe systemic infections [2]. In the United States, salmonellosis is the most common foodborne bacterial infection, accounting for 17.6 laboratory-confirmed illnesses per 100,000 persons annually [1,2]. More than 2,570 *Salmonella* serovars have been identified [1], yet, a limited number of these are responsible for most *Salmonella* infections in humans and domestic animals [2,3]. Cattle serve as a reservoir of *Salmonella*, mostly transmitted to humans through the fecal-oral route by consumption of contaminated food [2,3], or by direct contact with infected animals and the environment [4,5]. Dairy cattle, in particular, are an important component of the beef supply providing aproximately 5.7 billion pounds (22.7%) yearly [6], and likely contribute to environmental dissemination of *Salmonella*. The prevalence within infected herds ranges from <1 to 97% [7] with a variable level of colonization, shedding and persistence. Multiple factors influence prevalence of *Salmonella* in cattle, including season [8], geographical regions [9,10], and management practices [11,12]. Moreover, host-associated factors including parturition [13], stress caused by social interactions [14], or disruptions in the intestinal microbiome [15,16] may exacerbate the shedding of this zoonotic pathogen.

Gut microbial communities form a diverse ecosystem with a fundamental role in the host metabolic functions, and immunity [17]. However, disruptions in the microbial structure facilitate intestinal colonization of opportunistic enteric pathogens, including *Salmonella* [18]. To overcome the colonization resistance, *Salmonella* uses invasion strategies, the host inflammatory response and the lymphatic system machinery, for survival, proliferation [19] and systemic dissemination with a subsequent lymph node colonization [20,21]. In an inflamed intestine, *Salmonella* uses gene-regulated virulence mechanisms mainly guided by the type III secretion system [22] to compete with the gut commensals, and establish systemic and chronic infections [23,24]. Therefore, events associated with stress and/or disease may increase *Salmonella* shedding through disruptions in the gut microbiome. Investigations of the gut microbiome in food producing animals, mainly poultry and swine, have focused on effect of antibiotic usage, production practices, and diet modifications [25,26]. However, microbiome perturbations associated with parturition have not been investigated. Prior evidence has demonstrated that periparturient cattle are more likely to shed *Salmonella* [13]; nonetheless, associations between changes in the gut microbiome and exacerbation of fecal shedding of this pathogen during the periparturient period have not been longitudinally investigated. Thus, the objective of this longitudinal study was to characterize changes in the composition and diversity of the fecal microbiome of dairy cows during the periparturient period, and identify the association of such changes with the onset of *Salmonella* shedding. Improved understanding of the ecology of *Salmonella* within livestock reservoirs is critical for the development of strategies to prevent shedding and dissemination of this microorganism to the environment and between human and animal populations.

Materials and methods

Study design

This study was carried out in strict accordance with the recommendations in the Guide for the Care and Use of Laboratory Animals of the National Institutes of Health. The protocol was approved by The Ohio State University Institutional Animal Care and Use Committee (Animal Welfare Assurance Number A3261-0, Protocol Number 2013A00000099). This prospective cohort study in periparturient dairy cows was performed from August 2013 to January 2014. Although farm management practices are important potential interventions for *Salmonella* shedding, the focus of this manuscript was on cow-level changes in *Salmonella* shedding. A convenience sample of four free stall commercial dairy farms located within three hours of The Ohio State University were included. Among farms, a total of 98 non-lactating Holstein cows ("dry" cows) within 3 wks prior parturition were selected for the study, including 24 cows from farm A, 18 from farm B, 29 from farm C, and 27 from farm D. Expected parturition date was determined based on farm records of artificial insemination. Fecal samples were collected at four time points relative to the expected calving date; 3 wks (mean 21 d) and 1 wk (mean 7.2 d) prior to parturition, and at 1 wk (mean 7 d) and 3 wks (mean 24 d) post parturition.

The number of milking cows was 175 for farm A, 1,250 for farm B, 1,150 for farm C, and 1,250 for farm D. Farms B and D are owned by the same individuals. Farms A and C are closed herds that did not import any animals from outside the farm during the study period. In contrast, recently calved lactating heifers from outside sources were routinely imported onto both Farms B and D to be used as herd replacements. Sampling of imported heifers was not conducted in the present study. A *Salmonella* vaccine (*S*. Newport bacterial extract SRP cattle vaccine, Zoetis, Marysville, KS) was administered to all cows annually on farms B, C, and D and administered once per lactation on farm A. All cows from all farms were milked twice daily, housed in free stall pens on sand bedding during the "dry" (-60 to -1 days relative to calving, non-lactating stage) and "fresh" (recently calved cows, lactating stage) lactation periods, and were fed a total mixed ration that varied in composition according to the lactation stage. During the dry period, diets in all farms (with a few variations) consisted of alfalfa, grass hay, cracked corn, corn silage, and supplemented with selenium and vitamins A-D-E. Fresh cows were fed soybeans, corn silage, alfalfa hay, distillers grains, and cracked corn, supplemented with selenium, calcium, propylene glycol, and vitamins A-D-E. Specific components in all diets are provided in Table 1.

Sample collection

Approximately 10 g of feces was collected via rectal retrieval using a sterile plastic sleeve, immediately placed in a sterile bag (Nasco, Fort Atkinson, WI), transported to the laboratory

Table 1. Diet components among four dairy farms through the periparturient period.

Farm	Dry period (-3 weeks to calving)		Fresh period (calving to +3 weeks)	
	Solid component	Supplement	Solid component	Supplement
A	Alfalfa, grass hay, corn silage, cracked corn	Selenium	Corn silage, soybeans, distillers grains, cracked corn	Selenium, calcium, propylene glycol
B, D	Alfalfa haylage, grass hay, corn silage, soybeans, distillers grain, corn	Vitamins A-D-E, anionic salts, selenium, ionophores	Alfalfa haylage, corn silage, distillers grain	Vitamins A-D-E, selenium, ionophores
C	Corn silage, other silage, distiller grains	Vitamins A-D-E, anionic salts	Alfalfa haylage, corn silage, distillers grain, bakery bioproducts, corn	Vitamins A-D-E, propylene glycol

https://doi.org/10.1371/journal.pone.0196171.t001

on ice, and placed at 4˚C. Samples were processed within 24 hours for *Salmonella* culture and isolation, and the remaining fecal matter was stored at -80˚C until used for DNA extraction [27].

Salmonella isolation from bovine fecal samples

All fecal samples were subjected to a protocol for *Salmonella* culture and isolation using enrichment broths and selective media. Briefly, 4 g of feces were enriched into 36 mL tetrathionate broth (TTB) (BD, Spark, MD). After incubating at 37˚C for 18–24 hours, 0.1 mL TTB were pipetted into 10 mL Rappaport-Vassiliadis (RV) broth (BD, Spark, MD), and incubated at 42˚C for 18–24 hours. The following day, 10 μl RV was aseptically streaked out into xylose-lysine-tergitol-4 (XLT-4) agar plates (Remel, Lenexa, KS), followed by an overnight incubation at 37˚C. From each positive fecal sample, a single *Salmonella* colony was transferred to MacConkey agar (BD, Spark, MD) and identity confirmed by inoculation of the lactose-negative colonies onto a triple sugar iron slant, urea broth, and slide agglutination test using polyvalent and specific serogroup antisera (Cedarlane, Burlington, NC, USA).

Sample selection, DNA extraction, library preparation, and 16S rRNA gene sequencing

Out of the 98 periparturient cows, 63 calved within a week of their expected calving date, and thus had the four samples collected at -3, -1, +1, and +3 weeks relative to calving (WRC). Of those 63 cows, 48 were selected by simple random sampling for fecal microbiome analysis, including 8, 11, 20, and 9 cows from farms A, B, C and D, respectively. Subsequently, the total genomic DNA of the 192 samples (48 cows at 4 time points) was extracted. From each fecal sample, 0.2 g was used in a QIAamp Fast DNA Stool Mini Kit (Qiagen, Hilden, Germany) for total bacterial gDNA isolation, following the manufacturer's protocol. After extraction, DNA concentrations were measured using a NanoDrop spectrophotometer (NanoDrop8000, Thermo Fisher Scientific 8000, Delaware, USA). A minimum yield of 10 nmol gDNA was expected per sample, and a ratio of absorbance of ~1.8 at 260 nm and 280 nm was used to assess the purity of DNALibrary preparation was performed as previously described [28]. Briefly, conventional PCR was used to amplify the V4 hypervariable region of the *16S rRNA* gene using the 515F- 806R one way read barcoded primers (F5′-GTGCCAGCMGCCGCGGTAA-3′, R 5′-GGACTACHVGGGTWTCTAAT-3′). Each 25 μl PCR reaction contained 10 μl 5Prime Hot Master Mix (5Prime, Hilden, Germany) 0.5 μl (10pmol/μl) forward primer, 0.5 μl (10pmol/μl) reverse barcoded primer, 1 μl DNA template, and 13 μl of ultrapure PCR-grade water. Amplifications were performed in a MJMini thermocycler (PTC1148, Bio-Rad, Singapore) with an initial denaturation at 94˚C for 3 min, followed by 38 cycles of 94˚C for 45 sec, annealing at 55˚C for 1 min, extension at 72˚C for 90 sec, and final extension at 72˚C for 10 min. Amplicons of 300–350 bp were confirmed by gel electrophoresis, and concentrations were determined in a NanoDrop spectrophotometer. Two pools of 96 purified amplicons at equimolar concentrations of 240 ng per sample were submitted for next generation sequencing using the MiSeq Illumina platform (Miseq, Illumina, San Diego, CA) at the Ohio Agricultural Research and Development Center in Wooster, Ohio. For sequencing, each pool of 96 amplicons was mixed with a genomic library to generate diversity in a 40/60 ratio. A total of 10.7 pM of the mix were loaded into a MiSeq cartridge using the PE300 v3-600 cycles MiSeq kit. For sequencing data analysis, fastQ format files were retrieved from BaseSpace (Illumina, San Diego, CA, USA).

Sequencing analysis

High-throughput sequencing data were analyzed using mothur software package version 1.37.4 [29]. In mothur, contigs were built by the "make.contigs" function, and sequences with ambiguous bases and longer than desired length (maximum length 350 bp) were removed. After the pre-clustering step, residual singletons (cutoff = 1) were discarded using the "split.abund" function. Representative sequences were aligned to the V4 region of the SILVA rRNA database (release 109) [30,31]. UCHIME was used to remove chimeric sequences [32] and the RDP database (v9) was used to classify sequences at an 80% minimum pseudobootstrap confidence score [33,34]. Sequences belonging to chloroplasts, mitochondria, Archaea and Eukaryotes were removed by the "remove.lineage" function. Operational taxonomic units (OTUs) were classified with a 97% similarity and obtained by the "cluster.split" function. To evaluate community diversity, Shannon's diversity index was calculated using a fixed number of sequences per sample based on the lowest sequence number obtained by the "sub.sample" option in mothur.

Data analysis

SAS® version 9.4 (SAS Institute, Inc., Cary, NC) and the vegan package of R software version 3.3.1 (R Core Team, 2016) were used for statistical and bacterial diversity analysis, respectively. To evaluate the statistical differences in the prevalence of *Salmonella* among time points and farms, a logistic regression model was constructed using the *Salmonella* culture result (positive, negative) as the dichotomous response variable. The SAS model included weeks relative to parturition (-3, -1, 1, 3 wks), and farm (A, B, C, D) as fixed effects, and accounted for repeated sampling in time (REPEATED SUBJECTS statement).

For analysis of the fecal bacterial communities, normalization of abundance counts at different taxa levels was performed as previously described [35]. Briefly, all counts were transformed to a $\log_2$ value. The differences of each abundance count and the mean of all values were divided by the standard deviation of all values for that specific sample. Normalized taxonomy abundance counts were used to generate two dimensional principal component (PC) plots using a correlation matrix and heatmaps indicating the abundance and similarity of bacterial communities per sample. Dendrograms were constructed using the Yue and Clayton measure of dissimilarity (measure of community structure that includes share OTUs and relative abundances). PC plots, heatmaps and dendrograms were generated using the vegan package in R. Relative abundance at all taxa levels was calculated across samples. Taxa with low abundance (<0.1%) were grouped and analyzed as "Others". To evaluate the statistical differences in microbial composition according to farm, weeks relative to parturition, and *Salmonella* status, an analysis of similarities (ANOSIM) was implemented in R using the Jaccard dissimilarity distance with a permutation strategy.

Because changes in the microbiome were expected due to changes in diet and pen movements, the magnitude of changes in bacterial taxa in samples collected prior to parturition (-3 and -1 WRC) was compared between cows with *Salmonella* onset and non-onset. Cows with onset were those with a *Salmonella* negative status at -3 WRC and *Salmonella* positive status at -1 WRC. Cows with non-onset were those with a *Salmonella* negative status at -3 and -1 WRC. The differences in relative abundance between -3 and -1 WRC was calculated for the most abundant (>0.1%) bacterial phyla (n = 9) and families (n = 15). The differences in relative abundance were used to evaluate statistical differences between cows with or without the onset of *Salmonella* shedding using the Mann-Whitney non-parametric test. In addition, to evaluate if the relative abundance of predominant phyla at pre-calving differed from that at post-calving, the mean of relative abundances of -3 and -1 WRC was compared to the mean of those at

+3 and +1 WRC using the Wilcoxon signed-rank test. *P*-values <0.05 were considered to be statistically significant for all comparisons.

Results

Changes in *Salmonella* shedding through the periparturient period

A total of 98 periparturient cows were selected for this study. Out of those, 64% (63/98) calved within a week of their expected calving date. Detailed results on the changes in *Salmonella* shedding through the periparturient period have been presented elsewhere [36]. Briefly, 45.63% (115/252) samples were classified as *Salmonella* positive based on culture results. The proportion of cows shedding *Salmonella* significantly increased as cows experienced parturition (Fig 1). The prevalence of *Salmonella* in feces was significantly higher in the first week after parturition (55.5%) relative to three weeks prior to parturition (34.9%, odds ratio (OR) = 1.86, $p = 0.047$), and three weeks post-parturition (39.7%, OR = 1.8, $p = 0.004$). In addition, the prevalence at one week pre-parturition was significantly higher than the prevalence observed at three weeks after calving, 52.4% and 39.7%, respectively, (OR = 1.6, $p = 0.042$). The proportion of cows shedding *Salmonella* was also significantly different among farms ($p = 0.02$), with 38% (36/95) positive samples at farm A, 70% (50/71) at farm B, 31% (36/115) at farm C, and 42% (40/96) at farm D. This observation of shedding variation through the periparturient period and across farms was the reason to assess the changes in the composition, structure and diversity of fecal microbial communities associated with the onset of *Salmonella* shedding.

Sequencing metrics

Sequencing of 192 samples (48 cows at 4 time points) targeting the V4 region of the *16S rRNA* gene generated $3.2x10^7$ total reads (reads per sample mean = $1.7x10^5$, median = $1.68x10^5$, range $6.4x10^4$-$3.4x10^5$). After removal of ambiguous bases, homopolymers, chimeras and non-bacterial sequences, a subsample of 55,196 sequences were assigned into 3,319 OTUs clustered

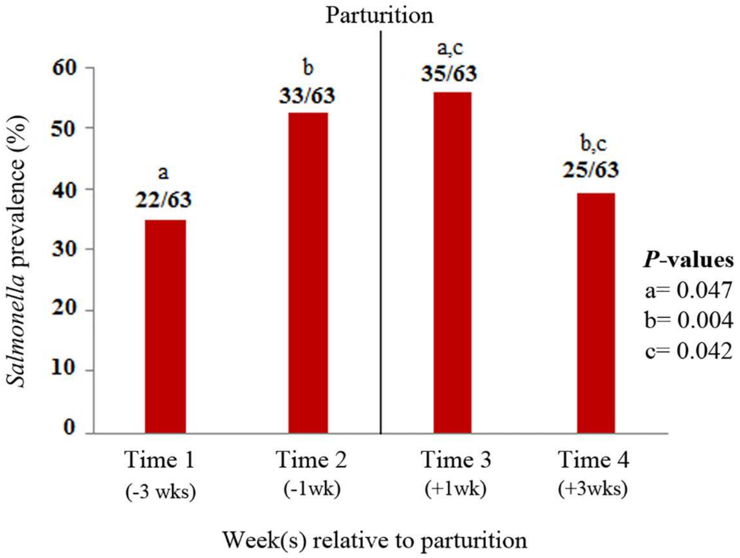

Fig 1. Prevalence of *Salmonella* during the periparturient period. Numbers on top of the bars indicate the proportion of *Salmonella* positive samples over the total sample number at each time point. Letters at the top of the figure represent the time points with a significantly different prevalence ($p<0.05$) according to the logistic regression analysis.

https://doi.org/10.1371/journal.pone.0196171.g001

at 97% sequence similarity. Consensus taxonomy for each OTU was used to generate phylotypes for each sample at 6 levels, from kingdom to genus (S1 Table). Fecal microbial communities of the 48 periparturient cows were classified into 26 phyla, 49 classes, 85 orders, 177 families, and 431 genera. From these, only 8 phyla, 17 classes, 24 families and 51 genera were identified as the "core fecal community" (taxa present in all samples).

Changes in fecal microbiome through the periparturient period

The overall fecal bacterial communities assessed from the -3 to +3 weeks relative to parturition were dominated by Bacteroidetes phylum (48.2%), followed by Firmicutes (42.4%), unclassified bacteria (4.3%) and Spirochaetes (2.4%). These four phyla accounted for 96.7% of the fecal bacterial population. Abundance variations in less dominant bacteria including Proteobacteria, Verrumicrobia, Euryarchaeota and bacteria that were not classified into a specific phylum (unclassified bacteria) were observed across samples. Within class-level taxa, the larger relative abundance was observed for Clostridia (47.5%) and Bacteroidia (32.4%), from Firmicutes and Bacteroidetes phyla, respectively. At family-level taxa, *Bacteroidaceae* was predominant over other bacteria (21.5%), followed by *Ruminococcaceae* (16.7%), *Clostridiaceae* (11.88%), and *Prevotellaceae* (4.75%). Normalized relative abundances at phylum and family level according to parturition period are depicted in Fig 2.

The sampling time point relative to parturition was significantly associated with changes in the structure of the fecal microbiome among the 48 periparturient cows (ANOSIM; $p<0.05$). The relative abundance of major bacterial phyla, with the exception of Bacteroidetes, significantly changed through this period ($p<0.05$). At post-calving, there was an observed increase ($p<0.001$) in the abundance of Spirochaetes and Actinobacteria, and a significantly lower abundance of Proteobacteria, Verrucomicrobia, and unclassified bacteria ($p<0.001$), compared to the pre-calving period (Table 2).

At family-level, there was a significant increase in the abundance of *Prevotellaceae*, *Lachnospiraceae*, and *Porphyromonadaceae*,; and a decrease of *Rumminococcaceae* post-parturition, which also corresponded to changes at genus-level, including an increase of *Treponema*, *Prevotella*, *and Clostridium_XI*, and a decrease of *Bacteroides*, respectively. Based on Shannon index, there was a significant increase in the diversity of the fecal microbiome at post-calving (2.8 vs 4.2, $p<0.04$), with a more diverse community at +3 WRC when compared to samples from the pre-calving period (-1 and -3 WRC; $p = 0.01$). In addition, PC plots revealed substantially different fecal bacterial communities between time points (Fig 3A), with a higher similarity

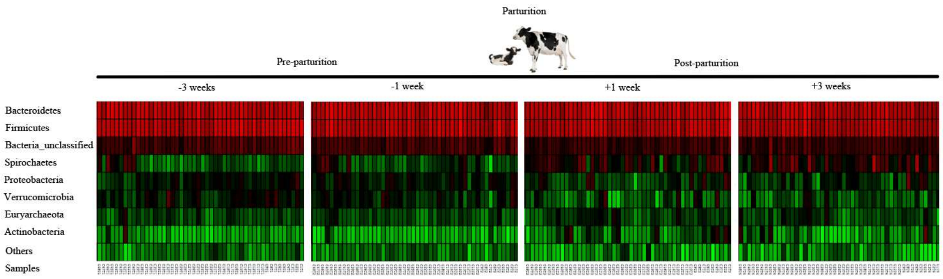

Fig 2. Phyla-level normalized relative abundance. Heatmap depicts the phyla normalized relative abundance of 192 samples collected at pre and post parturition periods. Red and green colors symbolize higher and lower relative abundances, respectively.

https://doi.org/10.1371/journal.pone.0196171.g002

Table 2. Relative abundance of bacterial phyla and families among 192 samples collected from 48 dairy cattle during the periparturient period.

Taxa	Overall relative abundances (%)	Pre-calving		Post-calving		*P*-value*
		Week -3	Week -1	Week +1	Week +3	
**Phylum**						
Bacteroidetes	47.80	45.44	48.35	48.31	49.17	0.16
Firmicutes	42.90	45.50	43.57	42.94	39.56	0.005
Bacteria_unclassified	4.38	5.17	4.73	3.54	4.07	<0.001
Spirochaetes	2.28	0.77	0.79	2.76	4.87	<0.001
Proteobacteria	0.94	1.03	1.05	0.78	0.93	<0.001
Verrucomicrobia	0.74	1.20	0.87	0.50	0.38	<0.001
Euryarchaeota	0.44	0.43	0.36	0.49	0.47	0.09
Actinobacteria	0.29	0.16	0.07	0.53	0.41	<0.001
Others	0.18	0.20	0.19	0.16	0.14	0.004
**Family**						
Bacteroidaceae	21.6	20.4	21.1	22.7	21.4	0.3
Ruminococcaceae	16.7	18.6	17.8	16.5	14.8	0.029
Clostridiaceae	11.9	13.2	12.8	11.2	11.3	0.006
Prevotellaceae	4.8	3.9	4.6	4.5	6.1	0.015
Bacteria_unclassified	4.3	5.2	4.7	3.5	4.1	<0.001
Lachnospiraceae	3.9	3.4	3.4	4.5	4.0	0.004
Spirochaetaceae	2.4	0.8	0.8	2.8	4.9	<0.001
Rikenellaceae	2.3	2.0	2.4	2.4	2.1	0.9
Porphyromonadaceae	0.9	0.7	0.8	1.1	1.1	<0.001

* *P*-values were calculated using the Wilcoxon signed-rank test that evaluated the differences in relative abundance at pre-calving compared to post-calving. *P*-values < 0.05 were considered significant.

https://doi.org/10.1371/journal.pone.0196171.t002

observed between -3 and -1 WRC compared to +1 and +3 WRC, primarily explained by the first component (38.9%, highest Eigenvalue).

Impact of the onset of *Salmonella* shedding on fecal microbiome

An increased relative abundance of Proteobacteria ($p = 0.02$), unclassified bacteria ($p = 0.008$) and phyla grouped as "Others" (Lentisphaerae, Fibrobacteres, Elusimicrobia, Tenericutes, TM7, Planctomycetes; $p = 0.04$) was observed in *Salmonella* positive samples. However, the diversity of microbial communities was not different between *Salmonella* positive and negative samples (4.3 vs 4.08, Shannon index, $p = 0.3$). A principal component analysis shows indistinguishable community clustering based on *Salmonella* status (Fig 4A).

The differences in bacterial abundance from cows that had an onset of *Salmonella* shedding were compared to cows that maintained a negative *Salmonella* status for the two samples prior to parturition. A numerically larger increase in Bacteroidetes, unclassified bacteria, Proteobacteria, Verrumicrobia, and Euryarchaeota, and a numerically larger decrease in Firmicutes were observed in cows with an onset of shedding (Fig 5). Non-significant differences, however, in population membership (Jaccard index, $p>0.1$), structure (Yue and Clayton, $p>0.05$), and diversity (Shannon, $p>0.06$) were identified between both microbial populations. A Fitch tree based on Yue and Clayton distances including the community structure and relative abundance (S1 Fig), and a bi-dimensional multivariate plot (Fig 4B) show indistinguishable fecal microbiomes between cows that had or did not have an onset of *Salmonella* shedding between -3 wks and -1 wk WRC.

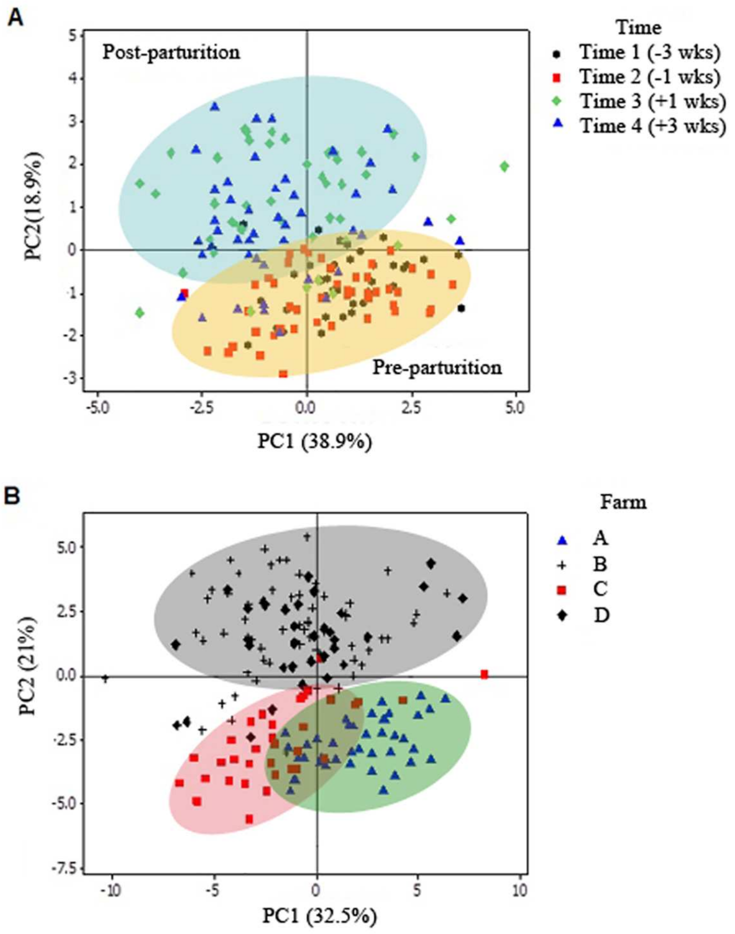

Fig 3. Bi-dimensional principal component plots comparing the total composition of the bacterial fecal microbiome of periparturient dairy cows. A) Fecal microbiome according to weeks relative to calving (-3, -1, +1, and +3 weeks). Elipses were drawn to highlight the similarity between bacterial communities at pre-parturition (yellow elipse) and post-parturition (light blue elipse). **B)** Fecal microbiome from farms A, B, C, and D. Large herds with an open heifer replacement system (Farms B and D, gray elipse) had a distinct clustering compared to those with in-farm replacement. Farm A (green elipse) had a significantly less diverse microbiome, indicated by Shannon index.

https://doi.org/10.1371/journal.pone.0196171.g003

Differences in the fecal microbiome between farms

Substantial differences in the microbiome were observed between farms. For instance, Bacteroidetes and unclassified genera from this phylum were dominant in the fecal samples from farms B, C, and D. Conversely, Firmicutes phylum and genera from Ruminococcaceae family (Firmicutes phylum) had the highest relative abundance in farm A. Both dominant phyla accounted for over 90% relative abundances in all farms. Analyses based on the Jaccard dissimilarity index indicated significant differences in Spirochaetes ($p = 0.038$), Proteobacteria ($p = 0.04$), Actinobacteria ($p<0.001$), and taxa with <0.1 abundance ($p<0.001$) among farms. Based on Shannon diversity index, the fecal bacterial community from farm A, managed in a closed replacement system and with fewer lactating cows, had significantly lower diversity than communities from farm B, C, and D (2.9 vs 4.38, 3.6, and 3.9, $p<0.01$). In addition, farm B, with open replacement system and with the largest herd size, showed higher diversity than farm C ($p = 0.01$). PC plots illustrated the difference in distribution of bacterial communities among farms, in which farm A had a distinct clustering compared to the others (Fig 3B).

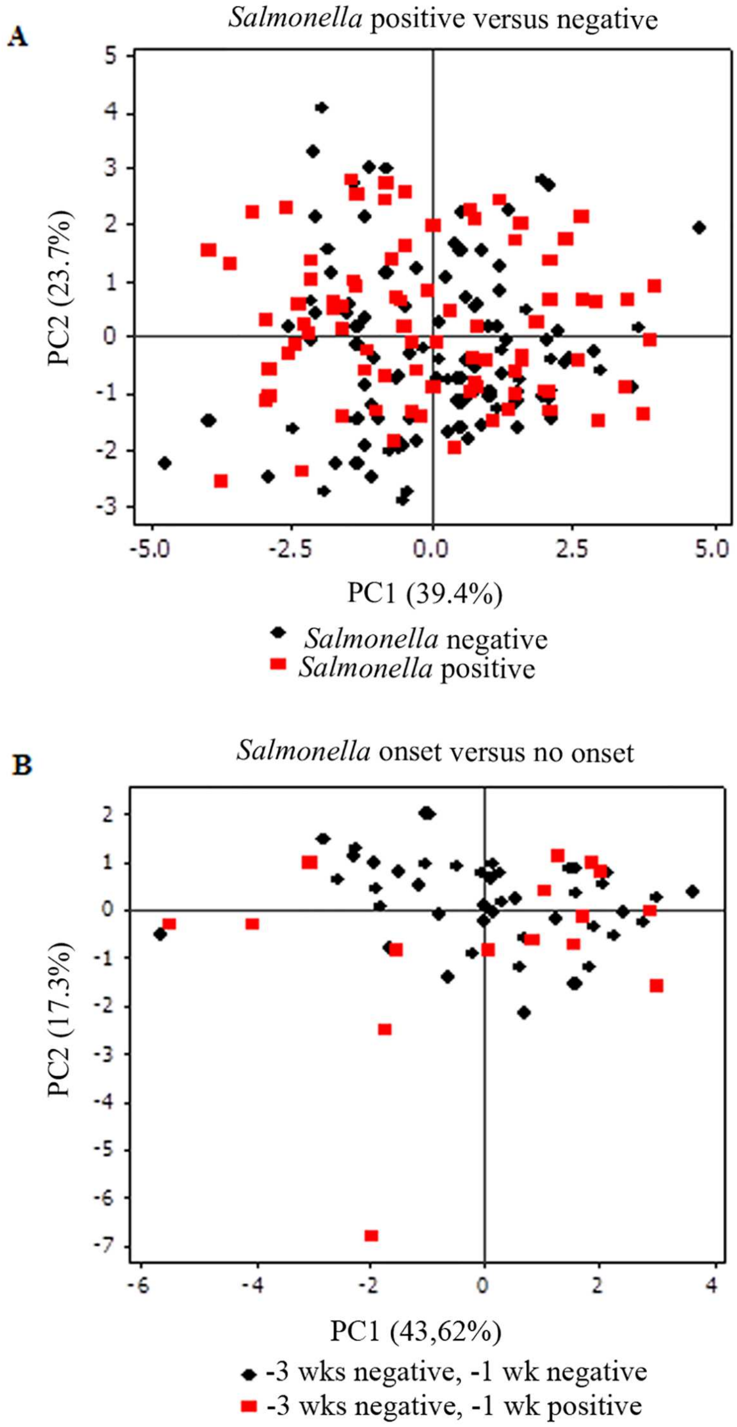

Fig 4. Bi-dimensional principal component plots representing the fecal microbiome of periparturient cattle based on *Salmonella* culture status. A) *Salmonella* culture positive (red squares) and negative samples (black circles). **B)** Cows that had an onset of *Salmonella* shedding between -3 and -1 weeks relative to parturition (red squares) compared to cows that remained with negative status (black circles). Non-significant differences were observed in diversity or communiy structure between fecal bacterial populations.

https://doi.org/10.1371/journal.pone.0196171.g004

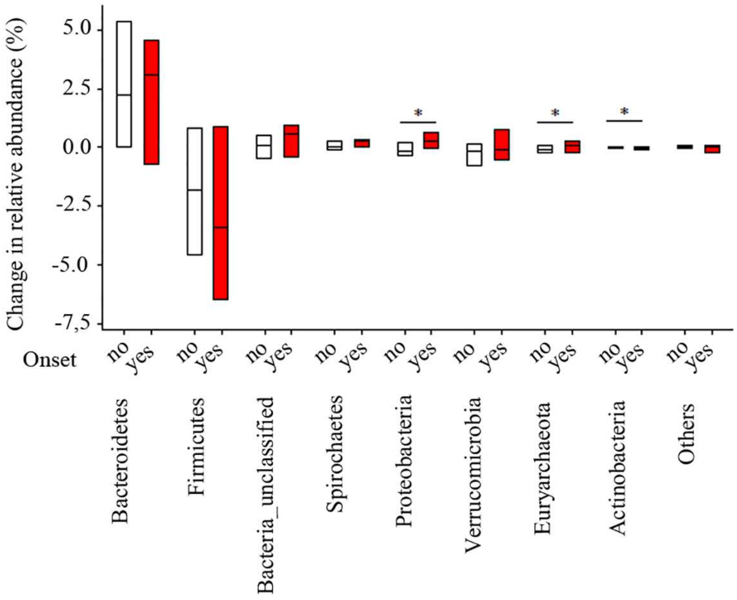

Fig 5. Effect of onset of *Salmonella* shedding on changes in fecal microbiome in dairy cattle between -3 and -1 weeks relative to parturition. Bacterial names at phyla-level and *Salmonella* onset status (yes, no) indicated on the *x*-axis. The box shows the range, and the included bar represents the median difference in bacterial phyla between both time points. Numerical differences were observed in most abundant phyla but non-statistical differences were detected. (*) represent significant changes in relative abundances ($p<0.05$).

https://doi.org/10.1371/journal.pone.0196171.g005

Effect of antimicrobial treatment on fecal microbiome

During the course of this study, 8 cows included for the microbiome analysis received antimicrobial therapies for treatment against mastitis (n = 2), metritis (n = 3), and an unknown cause (n = 3). Three of those cows were treated before the first sampling (-3 WRC), four cows were treated after parturition, and one cow before the last sampling. Drugs applied in sick animals had a broad antimicrobial spectrum, including 3rd generation cephalosporins (ceftiofur), and penicillins. Since antimicrobial therapies likely alter the gut microbiome [25–26], the impact of treatment on the abundance and diversity of the fecal microbiome was assessed. Results indicated a numerical decrease in the abundance of Firmicutes, unclassified bacteria and Proteobacteria in treated cows compared to non-treated animals. However, diminished abundances associated with antimicrobial therapy were only statistically significant for Verrumicrobia ($p = 0.001$), Euryarchaeota ($p = 0.04$), and Actinobacteria ($p = 0.01$). Exclusion of treated animals from the analysis of time, *Salmonella* status, and farm was performed; nevertheless, this exclusion did not affect the associations reported above.

Discussion

Next-generation sequencing of the V4 hypervariable region of the *16S rRNA* gene was used in the present study to identify the fecal microbiome in periparturient cattle and to assess changes in these fecal microbial communities associated with the onset of *Salmonella*. The results presented here are consistent with previous research that showed Bacteroidetes and Firmicutes as the two most dominant phyla during the periparturient period, accounting for over 90% of the total bacterial population [16]. In accordance with other studies, these two phyla owned also the highest relative abundance in calves and non-periparturient lactating cows [37–39]. We observed significant changes in the relative abundance of some phyla that accounted for less

than 10% of the total bacterial population at the time relative to parturition, including Spirochaetes, Proteobacteria, Verrucomicrobia, and Actinobacteria. Differences in these taxa could be attributed to diet changes or pen movement [40,41] since cows were fed a different diet during the transition from the"dry" to "lactating" stage, and were moved from free stall to fresh cow pens after parturition.

Factors related to feeding and management practices have been described as determinants for the hindgut microbial structure [41,42]. Results presented in this study indicated strongly distinct fecal microbial community composition, diversity and abundance between dairy cattle populations. The farm with a smaller herd size and managed with a closed heifer replacement system showed lower microbiome diversity and different clustering in the multivariate analysis, compared to larger farms that routinely imported animals, nonetheless, the ability to extrapolate these observations to larger population of farms is limited since only four farms were evaluated in this prospective cohort studyLarger studies with more herds are necessary to determine if other factors including herd size or heifer replacement systems could be associated with bacterial diversity. Although diet composition was formulated similarly between the farms included in this study, the relative proportion of ingredients was not provided and may have impacted the microbiome. However, since we controlled statistically for farm, this should not have affected our between-cow analysis. Further research about the interaction that specific compounds, diet formulation, or administration of beneficial microorganisms have on *Salmonella* colonization is recommended, since understanding the microbiome may lead to diet interventions on farms.

Disruptions in the gastrointestinal microbiome have been associated with diseases in animals in a variety of studies. Higher abundance of Actinobacteria, and Bacteroidetes has been identified in fecal samples of cattle colonized by *Mycobacterium avium* subsp. *paratuberculosis* [38] and *Campylobacter jejuni* [37], respectively. In pigs, increased abundance of Actinobacteria in the colon has also been associated with colonization of *Lawsonia intracellularis* [43]. Colic development has been observed in pregnant mares with higher relative abundances of Firmicutes and Proteobacteria [44]. In food-producing animals, however, the effect of *Salmonella* on the gut microbiome composition is still not clear as many factors can alter the mechanisms related to colonization. Some studies have reported significant microbiome variations in the presence of *Salmonella* [45], including a Proteobacteria bloom associated with microbiome dysbiosis [18,46]. However, others have not been able to associate changes in specific bacterial species with the exacerbation of *Salmonella* colonization in cattle [47] or in older chickens infected with *Salmonella enteritidis* [48].

Despite the fact that an increased abundance of some bacteria taxa was observed in *Salmonella* positive samples, results obtained in the present study found no differences in overall fecal microbial diversity and structure associated with the onset of *Salmonella* shedding. Similarly, Haley and colleages [49] could not identify differences in microbial communities of fecal grab samples from *Salmonella* positive lactating cows compared to negative ones, and as herein, the community membership was indistinguishable between shedders and non-shedders. In dairy cattle, the composition, diversity and structure of microbiota vary among the intestinal segments [15], and it has been observed that bacterial communities from intestinal mucosa may vary from those present in the digesta [50]. Thus, fecal samples may not have captured disruptions in the microbial community at other locations in the gastrointestinal tract that could have influenced *Salmonella* shedding. Nonetheless, the sampling method in this study was sufficient to demonstrate differences across time and between farms. The increased prevalence of *Salmonella* in feces around parturition as observed herein is concordant with previous reports in other animal species [51]. Yet, this study demonstrated an onset of shedding prior to parturition that was unexplained by changes in the fecal microbiome.

Conclusions

This study demonstrates that cattle fecal microbiome significantly changes with parturition, with a significant increase in bacterial diversity after calving. Additionally, the diversity and structure of the fecal microbial community is distinct between farms. Overall, cows enrolled in this study had a significant increase in *Salmonella* shedding prior to parturition, but the onset of shedding was not associated with changes in the structure or diversity of the fecal microbiome. Since this study evaluated the presence or absence of *Salmonella* in feces, further research should assess the temporal changes in microbiota associated with fecal concentrations of *Salmonella* in periparturient cattle.

Supporting information

S1 Fig. Fitch tree of distance estimates between cows that had an onset (black) or non-onset (red) of *Salmonella* shedding between -3 and -1 weeks relative to parturition. Non distinct clusters between bacterial communities of cows that had onset and non-onset were observed.
(PDF)

S1 Table. Fecal bacterial taxa from 48 periparturient dairy cows at four time points.
(XLSX)

S2 Table. Genus level microbiome composition at 3-, -1, +1, and +3 weeks relative to parturition in *Salmonella* positive and negative dairy cows at four different farms.
(XLSX)

Acknowledgments

The authors would like to thank the farm owners and managers for the willingness to participate in this study, and to Dr. Santiago Bas, and Dr. Adrian Barragan, for their cooperation during the sample collection.

Author Contributions

Conceptualization: Lohendy Muñoz-Vargas.

Data curation: Lohendy Muñoz-Vargas.

Formal analysis: Lohendy Muñoz-Vargas, Stephen O. Opiyo.

Funding acquisition: Gregory Habing.

Investigation: Lohendy Muñoz-Vargas, Rose Digianantonio, Michele L. Williams, Gregory Habing.

Methodology: Lohendy Muñoz-Vargas, Rose Digianantonio, Michele L. Williams, Asela Wijeratne, Gregory Habing.

Project administration: Gregory Habing.

Resources: Gregory Habing.

Software: Lohendy Muñoz-Vargas, Stephen O. Opiyo, Asela Wijeratne.

Supervision: Gregory Habing.

Validation: Lohendy Muñoz-Vargas.

Visualization: Lohendy Muñoz-Vargas.

Writing – original draft: Lohendy Muñoz-Vargas.

Writing – review & editing: Stephen O. Opiyo, Rose Digianantonio, Michele L. Williams, Asela Wijeratne, Gregory Habing.

References

1. Grimont P, Weill F-X. Antigenic formulae of the Salmonella serovars. 9TH Edition. WHO Collaborating Centre for Reference and Research on Salmonella; 2007.
2. CDC. Multistate Outbreak of Salmonella Typhimurium Infections Linked to Ground Beef. Centers for Disease Control and Prevention; 2013.
3. Meyer C, Thiel S, Ullrich U, Stolle A. Salmonella in raw meat and by-products from pork and beef. J Food Prot. 2010 Oct; 73(10):1780–4. PMID: 21067664
4. CDC. Multistate Outbreak of Multidrug-Resistant Salmonella Heidelberg Infections Linked to Contact with Dairy Bull Calves. Centers for Disease Control and Prevention; 2016.
5. Cummings KJ, Warnick LD, Davis MA, Eckmann K, Gröhn YT, Hoelzer K, et al. Farm animal contact as risk factor for transmission of bovine-associated Salmonella subtypes. Emerg Infect Dis. 2012 Dec; 18 (12):1929–36. https://doi.org/10.3201/eid1812.110831 PMID: 23171627
6. Boetl B. In The Cattle Markets: Dairy Cattle Impact on Beef Supplies. Dairy Herd Management; 2017 Oct 31.
7. Huston CL, Wittum TE, Love BC, Keen JE. Prevalence of fecal shedding of Salmonella spp in dairy herds. J Am Vet Med Assoc. 2002 Mar 1; 220(5):645–9. PMID: 12418525
8. Edrington TS, Ross TT, Callaway TR, Martinez CH, Hume ME, Genovese KJ, et al. Investigation into the seasonal salmonellosis in lactating dairy cattle. Epidemiol Infect. 2008 Mar; 136(3):381–90. https://doi.org/10.1017/S0950268807008680 PMID: 17506921
9. Lewerin SS, Skog L, Frössling J, Wahlström H. Geographical distribution of salmonella infected pig, cattle and sheep herds in Sweden 1993–2010. Acta Vet Scand. 2011 Oct 5; 53(1):51.
10. Strawn LK, Danyluk MD, Worobo RW, Wiedmann M. Distributions of Salmonella Subtypes Differ between Two U.S. Produce-Growing Regions. Appl Environ Microbiol. 2014 Jul; 80(13):3982–91. https://doi.org/10.1128/AEM.00348-14 PMID: 24747908
11. Habing GG, Lombard JE, Kopral CA, Dargatz DA, Kaneene JB. Farm-Level Associations with the Shedding of *Salmonella* and Antimicrobial-Resistant *Salmonella* in U.S. Dairy Cattle. Foodborne Pathog Dis. 2012 Sep; 9(9):815–21. https://doi.org/10.1089/fpd.2012.1149 PMID: 22870913
12. Manyi-Loh CE, Mamphweli SN, Meyer EL, Makaka G, Simon M, Okoh AI. An Overview of the Control of Bacterial Pathogens in Cattle Manure. Int J Environ Res Public Health [Internet]. 2016 Sep [cited 2016 Dec 12]; 13(9). Available from: http://www.ncbi.nlm.nih.gov/pmc/articles/PMC5036676/
13. Fossler CP, Wells SJ, Kaneene JB, Ruegg PL, Warnick LD, Bender JB, et al. Herd-level factors associated with isolation of Salmonella in a multi-state study of conventional and organic dairy farms II. Salmonella shedding in calves. Prev Vet Med. 2005 Sep 12; 70(3–4):279–91. https://doi.org/10.1016/j.prevetmed.2005.04.002 PMID: 15963584
14. Proudfoot KL, Veira DM, Weary DM, von Keyserlingk MAG. Competition at the feed bunk changes the feeding, standing, and social behavior of transition dairy cows. J Dairy Sci. 2009 Jul; 92(7):3116–23. https://doi.org/10.3168/jds.2008-1718 PMID: 19528589
15. Frey JC, Pell AN, Berthiaume R, Lapierre H, Lee S, Ha JK, et al. Comparative studies of microbial populations in the rumen, duodenum, ileum and faeces of lactating dairy cows. J Appl Microbiol. 2010 Jun; 108(6):1982–93. https://doi.org/10.1111/j.1365-2672.2009.04602.x PMID: 19863686
16. Pitta DW, Kumar S, Vecchiarelli B, Shirley DJ, Bittinger K, Baker LD, et al. Temporal dynamics in the ruminal microbiome of dairy cows during the transition period. J Anim Sci. 2014 Sep; 92(9):4014–22. https://doi.org/10.2527/jas.2014-7621 PMID: 25023800
17. Round JL, Mazmanian SK. The gut microbiome shapes intestinal immune responses during health and disease. Nat Rev Immunol. 2009 May; 9(5):313–23. https://doi.org/10.1038/nri2515 PMID: 19343057
18. Ahmer BMM, Gunn JS. Interaction of Salmonella spp. with the Intestinal Microbiota. Front Microbiol [Internet]. 2011 [cited 2016 Apr 21]; 2. Available from: http://journal.frontiersin.org/article/10.3389/fmicb.2011.00101/abstract
19. Stecher B, Robbiani R, Walker AW, Westendorf AM, Barthel M, Kremer M, et al. Salmonella enterica Serovar Typhimurium Exploits Inflammation to Compete with the Intestinal Microbiota. PLoS Biol

[Internet]. 2007 Oct [cited 2017 Jan 12]; 5(10). Available from: http://www.ncbi.nlm.nih.gov/pmc/articles/PMC1951780/

20. Andino A, Hanning I. Salmonella enterica: Survival, Colonization, and Virulence Differences among Serovars. Sci World J [Internet]. 2015 [cited 2017 Jan 4]; 2015. http://www.ncbi.nlm.nih.gov/pmc/articles/PMC4310208/
21. Muñoz-Vargas L, Finney SK, Hutchinson H, Masterson MA, Habing G. Impact of Clinical Salmonellosis in Veal Calves on the Recovery of Salmonella in Lymph Nodes at Harvest. Foodborne Pathog Dis. 2017 Nov; 14(11):678–85. https://doi.org/10.1089/fpd.2017.2303 PMID: 28910140
22. Cardenal-Muñoz E, Ramos-Morales F. Analysis of the Expression, Secretion and Translocation of the Salmonella enterica Type III Secretion System Effector SteA. PLoS ONE [Internet]. 2011 Oct 27 [cited 2017 Jan 12]; 6(10). Available from: http://www.ncbi.nlm.nih.gov/pmc/articles/PMC3203157/
23. Santos RL. Pathobiology of Salmonella, Intestinal Microbiota, and the Host Innate Immune Response. Front Immunol [Internet]. 2014 May 26 [cited 2016 Apr 21]; 5. Available from: http://journal.frontiersin.org/article/10.3389/fimmu.2014.00252/abstract
24. Behnsen J, Perez-Lopez A, Nuccio S-P, Raffatellu M. Exploiting host immunity: the Salmonella paradigm. Trends Immunol. 2015 Feb; 36(2):112–20. https://doi.org/10.1016/j.it.2014.12.003 PMID: 25582038
25. Zhang C, Yu M, Yang Y, Mu C, Su Y, Zhu W. Effect of early antibiotic administration on cecal bacterial communities and their metabolic profiles in pigs fed diets with different protein levels. Anaerobe. 2016 Dec; 42:188–96. https://doi.org/10.1016/j.anaerobe.2016.10.016 PMID: 27816724
26. Baron S, Jouy E, Touzain F, Bougeard S, Larvor E, de Boisseson C, et al. Impact of the administration of a third-generation cephalosporin (3GC) to one-day-old chicks on the persistence of 3GC-resistant Escherichia coli in intestinal flora: An in vivo experiment. Vet Microbiol. 2016 Mar 15; 185:29–33. https://doi.org/10.1016/j.vetmic.2016.01.020 PMID: 26931388
27. Vogtmann E, Chen J, Amir A, Shi J, Abnet CC, Nelson H, et al. Comparison of Collection Methods for Fecal Samples in Microbiome Studies. Am J Epidemiol. 2017 Jan 15; 185(2):115–23. https://doi.org/10.1093/aje/kww177 PMID: 27986704
28. Caporaso JG, Lauber CL, Walters WA, Berg-Lyons D, Huntley J, Fierer N, et al. Ultra-high-throughput microbial community analysis on the Illumina HiSeq and MiSeq platforms. ISME J. 2012; 6(8):1621–1624. https://doi.org/10.1038/ismej.2012.8 PMID: 22402401
29. Schloss PD, Westcott SL, Ryabin T, Hall JR, Hartmann M, Hollister EB, et al. Introducing mothur: open-source, platform-independent, community-supported software for describing and comparing microbial communities. Appl Environ Microbiol. 2009 Dec; 75(23):7537–41. https://doi.org/10.1128/AEM.01541-09 PMID: 19801464
30. Pruesse E, Quast C, Knittel K, Fuchs BM, Ludwig W, Peplies J, et al. SILVA: a comprehensive online resource for quality checked and aligned ribosomal RNA sequence data compatible with ARB. Nucleic Acids Res. 2007; 35(21):7188–96. https://doi.org/10.1093/nar/gkm864 PMID: 17947321
31. Quast C, Pruesse E, Yilmaz P, Gerken J, Schweer T, Yarza P, et al. The SILVA ribosomal RNA gene database project: improved data processing and web-based tools. Nucleic Acids Res. 2013 Jan; 41 (Database issue):D590–596. https://doi.org/10.1093/nar/gks1219 PMID: 23193283
32. Edgar RC, Haas BJ, Clemente JC, Quince C, Knight R. UCHIME improves sensitivity and speed of chimera detection. Bioinforma Oxf Engl. 2011 Aug 15; 27(16):2194–200.
33. Wang Q, Garrity GM, Tiedje JM, Cole JR. Naive Bayesian classifier for rapid assignment of rRNA sequences into the new bacterial taxonomy. Appl Environ Microbiol. 2007 Aug; 73(16):5261–7. https://doi.org/10.1128/AEM.00062-07 PMID: 17586664
34. Wang D, Hutson AD. A Smooth Bootstrap Procedure towards Deriving Confidence Intervals for the Relative Risk. Commun Stat Theory Methods. 2014; 43(9):1979–90. https://doi.org/10.1080/03610926.2012.681418 PMID: 26997746
35. Zhao W, Wang Y, Liu S, Huang J, Zhai Z, He C, et al. The Dynamic Distribution of Porcine Microbiota across Different Ages and Gastrointestinal Tract Segments. Li X, editor. PLOS ONE. 2015 Feb 17; 10(2):e0117441. https://doi.org/10.1371/journal.pone.0117441 PMID: 25688558
36. Digianantonio R. [MPH thesis]. [Columbus, Ohio]: The Ohio State University; 2015.
37. Dong H-J, Kim W, An J-U, Kim J, Cho S. The Fecal Microbial Communities of Dairy Cattle Shedding Shiga Toxin-Producing Escherichia coli or Campylobacter jejuni. Foodborne Pathog Dis. 2016 Sep; 13(9):502–8. https://doi.org/10.1089/fpd.2016.2121 PMID: 27385033
38. Fecteau M-E, Pitta DW, Vecchiarelli B, Indugu N, Kumar S, Gallagher SC, et al. Dysbiosis of the Fecal Microbiota in Cattle Infected with Mycobacterium avium subsp. paratuberculosis. PloS One. 2016; 11(8):e0160353. https://doi.org/10.1371/journal.pone.0160353 PMID: 27494144

39. Klein-Jöbstl D, Schornsteiner E, Mann E, Wagner M, Drillich M, Schmitz-Esser S. Pyrosequencing reveals diverse fecal microbiota in Simmental calves during early development. Front Microbiol. 2014; 5:622. https://doi.org/10.3389/fmicb.2014.00622 PMID: 25452753

40. David LA, Maurice CF, Carmody RN, Gootenberg DB, Button JE, Wolfe BE, et al. Diet rapidly and reproducibly alters the human gut microbiome. Nature. 2014 Jan 23; 505(7484):559–63. https://doi.org/10.1038/nature12820 PMID: 24336217

41. Shanks OC, Kelty CA, Archibeque S, Jenkins M, Newton RJ, McLellan SL, et al. Community Structures of Fecal Bacteria in Cattle from Different Animal Feeding Operations. Appl Environ Microbiol. 2011 May 1; 77(9):2992–3001. https://doi.org/10.1128/AEM.02988-10 PMID: 21378055

42. Callaway TR, Dowd SE, Edrington TS, Anderson RC, Krueger N, Bauer N, et al. Evaluation of bacterial diversity in the rumen and feces of cattle fed different levels of dried distillers grains plus solubles using bacterial tag-encoded FLX amplicon pyrosequencing. J Anim Sci. 2010 Dec; 88(12):3977–83. https://doi.org/10.2527/jas.2010-2900 PMID: 20729286

43. Borewicz KA, Kim HB, Singer RS, Gebhart CJ, Sreevatsan S, Johnson T, et al. Changes in the Porcine Intestinal Microbiome in Response to Infection with Salmonella enterica and Lawsonia intracellularis. PloS One. 2015; 10(10):e0139106. https://doi.org/10.1371/journal.pone.0139106 PMID: 26461107

44. Weese JS, Holcombe SJ, Embertson RM, Kurtz KA, Roessner HA, Jalali M, et al. Changes in the faecal microbiota of mares precede the development of post partum colic. Equine Vet J. 2015 Nov 1; 47(6):641–9. https://doi.org/10.1111/evj.12361 PMID: 25257320

45. Drumo R, Pesciaroli M, Ruggeri J, Tarantino M, Chirullo B, Pistoia C, et al. Salmonella enterica Serovar Typhimurium Exploits Inflammation to Modify Swine Intestinal Microbiota. Front Cell Infect Microbiol. 2015; 5:106. https://doi.org/10.3389/fcimb.2015.00106 PMID: 26835435

46. Shin N-R, Whon TW, Bae J-W. Proteobacteria: microbial signature of dysbiosis in gut microbiota. Trends Biotechnol. 2015 Sep; 33(9):496–503. https://doi.org/10.1016/j.tibtech.2015.06.011 PMID: 26210164

47. Patton TG, Scupham AJ, Bearson SMD, Carlson SA. Characterization of fecal microbiota from a Salmonella endemic cattle herd as determined by oligonucleotide fingerprinting of rDNA genes. Vet Microbiol. 2009 May 12; 136(3–4):285–92. https://doi.org/10.1016/j.vetmic.2008.10.032 PMID: 19091494

48. Juricova H, Videnska P, Lukac M, Faldynova M, Babak V, Havlickova H, et al. Influence of Salmonella enterica Serovar Enteritidis Infection on the Development of the Cecum Microbiota in Newly Hatched Chicks. Appl Environ Microbiol. 2013 Jan 15; 79(2):745–7. https://doi.org/10.1128/AEM.02628-12 PMID: 23144133

49. Haley BJ, Pettengill J, Gorham S, Ottesen A, Karns JS, Van Kessel JAS. Comparison of Microbial Communities Isolated from Feces of Asymptomatic Salmonella-Shedding and Non-Salmonella Shedding Dairy Cows. Front Microbiol [Internet]. 2016 Jun 1 [cited 2016 Nov 7]; 7. Available from: http://www.ncbi.nlm.nih.gov/pmc/articles/PMC4887466/

50. Mao S, Zhang M, Liu J, Zhu W. Characterising the bacterial microbiota across the gastrointestinal tracts of dairy cattle: membership and potential function. Sci Rep. 2015 Nov 3; 5:srep16116.

51. Nollet N, Houf K, Dewulf J, De Kruif A, De Zutter L, Maes D. Salmonella in sows: a longitudinal study in farrow-to-finish pig herds. Vet Res. 2005 Aug; 36(4):645–56. https://doi.org/10.1051/vetres:2005022 PMID: 15955287
